# Acceleration–Deceleration Match Demands Relative to Sprint Capacity in Cerebral Palsy Football: An Exploratory Demand–Capacity Analysis

**DOI:** 10.3390/s26216881

**Published:** 2026-10-30

**Authors:** Iván Peña-González, Alba Roldán, Alejandro Javaloyes, Manuel Moya-Ramón

**Affiliations:** 1 Sports Research Centre, Universidad Miguel Hernández de Elche, Avda. de La Universidad S/N, 03202 Elche, Spain; ipena@umh.es (I.P.-G.); ajavaloyes@umh.es (A.J.); mmoya@umh.es (M.M.-R.); 2 Spanish Federation of Sports for People with Cerebral Palsy and Acquired Brain Injury (FEDPC), 28011 Madrid, Spain

**Keywords:** CP football, Para football, external load, accelerations, ratio scaling, mixed models, sport classification

## Abstract

Interpreting match load relative to individual capacity may be particularly relevant in cerebral palsy (CP) football, where impairment affects both capacity and match output. This exploratory study examined how the distance covered during high-intensity acceleration and deceleration (ACCDEC) relates to acceleration capacity (average 5-m sprint velocity, V5), whether the ratio ACCDEC/V5 meets the conditions for ratio scaling, and whether ACCDEC and ACCDEC/V5 differ between sport classes. GNSS/IMU match data (273 player–match observations; 63 male players; seven Spanish National League teams) were analysed with linear mixed-effects models. Between players, ACCDEC/V5 was nearly identical to ACCDEC (r = 0.97) and uncorrelated with V5 (r = 0.08), and the ACCDEC–V5 relationship was compatible with proportionality (intercept −0.52 m·min^−1^; 95% CI −4.06 to 3.01). Higher V5 was associated with greater ACCDEC (B = 0.91 m·min^−1^ per m·s^−1^; *p* = 0.029). ACCDEC tended to be lower in FT1 than in FT3 players (*p* = 0.056), whereas ACCDEC/V5 did not differ between classes (*p* = 0.304); neither class effect was robust in sensitivity analyses. ACCDEC/V5 is a capacity-scaled descriptor of match demands rather than a measure of physiological cost, and its added value for load monitoring and classification remains to be established.

## 1. Introduction

Cerebral palsy (CP) football is a high-intensity intermittent team Para sport that requires players to perform repeated accelerations, decelerations, changes in direction, and short-duration high-speed actions interspersed with sustained low- and moderate-intensity activity. Despite the adaptations to rules compared to mainstream football, match-play demands are underpinned by the concurrent contribution of aerobic and anaerobic energy systems, while being strongly modulated by neurological impairments that affect motor control, balance, coordination, and force production [1,2]. Time–motion and inertial sensor analyses have shown that competitive matches impose substantial external loads, characterized by frequent neuromuscular actions and considerable volumes of moderate- and high-intensity running, although absolute values remain lower than those reported in mainstream football [3]. Importantly, match demands are highly sensitive to contextual factors such as competitive level and opponent quality, with international competitions eliciting markedly greater locomotor and mechanical outputs than national-level contexts [4,5,6]. Additionally, physical demands are shaped by the classification system in CP football, which allocates players to three sport classes (FT1: high impairment, FT2: moderate impairment, and FT3: mild impairment) according to the impact of impairment on football-specific performance. This classification system has direct implications for game dynamics, as competition regulations require each team to field at least one FT1 player at all times and allow no more than one FT3 player on the field [7]. As a result, teams must continuously balance players with heterogeneous functional profiles, which directly influences tactical organisation, role allocation, and the distribution of physical and neuromotor demands across players. Consistent with this regulatory and functional framework, players with milder impairments consistently achieve higher peak velocities, greater high-intensity running distances, and increased acceleration and deceleration frequencies compared to those with more severe impairments [8,9]. Together, these findings highlight the complex interaction between impairment-related constraints and game dynamics, emphasizing the need for classification- and context-specific approaches to load monitoring and training prescription in CP football.

In both mainstream and CP football, a substantial body of scientific literature has focused on the quantification of external match and training demands, enabling a detailed description of locomotor actions such as total distance covered, running velocity, and the frequency and magnitude of accelerations and decelerations [10,11]. The widespread use of wearable technologies has markedly improved the objectivity and resolution of these measurements, thereby contributing substantially to the analysis of physical performance in competitive contexts. However, external load metrics recorded during match play should not be interpreted per se as direct indicators of sporting performance, as they are strongly influenced by a wide range of contextual variables, including tactical organization, playing position, opposition quality, match status, and competitive level [10,12,13].

Moreover, growing evidence indicates that external load data, when interpreted in isolation, provide an incomplete representation of the player’s response to match demands, as comparable external workloads may be associated with different internal responses across players [14]. Previous research has shown moderate relationships between external load metrics and markers of internal load, such as heart rate responses or ratings of perceived exertion [14]. This variability reflects the imperfect relationship between external and internal load measures and is influenced by individual physical capacity, functional profile, and tolerance to effort. Consequently, a comprehensive interpretation of match demands requires integrating external load measures with contextual and individual-specific factors. This need becomes particularly relevant in CP football, where impairment-related constraints further modulate the relationship between physical output and performance [5,15]. In this context, several approaches have been proposed to individualise external load, such as speed or acceleration thresholds expressed relative to each player’s maximal capacities, acceleration–speed profiles, or match outputs relative to peak locomotor demands [16,17,18]. These approaches individualise the thresholds used to quantify load, but they do not express the resulting match load relative to a measure of the player’s capacity.

Interpreting match load relative to each player’s capacity is particularly relevant in Para sport contexts such as CP football, a modality characterised by marked functional heterogeneity within and across sport classes [19,20]. Differences in motor control, strength, coordination, and acceleration capacity directly influence the ability to perform high-intensity actions during competition [9,21], and recent studies have characterised match demands, their association with field-based tests, and positional and perceived-exertion responses in CP football [22,23,24,25], as well as the activity-limitation tests that best predict sport class [26]. Notably, sprint tests have shown limited associations with match variables in international CP footballers [24], so the extent to which match demands scale with acceleration capacity remains unclear. A simple way to express match demands relative to capacity, applicable when only routinely collected sprint tests and match reports are available, is to divide match load by a capacity measure, yielding a demand–capacity ratio. Ratios, however, are only appropriate when the numerator is proportional to the denominator (i.e., when their relationship passes through the origin); otherwise, they do not remove the influence of the denominator and can generate spurious correlations and group differences [27,28,29]. To our knowledge, no study has examined demand–capacity ratios in CP football or tested whether they meet these conditions.

Therefore, the aims of this exploratory study were: (i) to examine the association between the distance covered during high-intensity acceleration and deceleration (ACCDEC) and short-distance acceleration capacity (average 5-m sprint velocity, V5) in CP football players; (ii) to examine whether a ratio index (ACCDEC/V5) meets the conditions for ratio scaling and what information it provides beyond its components; and (iii) to compare ACCDEC and ACCDEC/V5 between sport classes while accounting for repeated observations of the same players. Rather than hypotheses that would be confirmed arithmetically by the construction of the index, we examined the following falsifiable expectations: (a) players with higher V5 would accumulate more ACCDEC per minute of play; (b) the ACCDEC–V5 relationship would be approximately proportional, so that ACCDEC/V5 would be uncorrelated with V5; and (c) ACCDEC would be lower in players with greater impairment (FT1), and these class differences would be attenuated when ACCDEC was expressed relative to, or adjusted for, V5.

## 2. Materials and Methods

### 2.1. Study Design

This observational study examined acceleration–deceleration match demands relative to individual sprint capacity in CP football under real competitive conditions. Sprint testing was conducted before the start of the competitive season, to characterise individual acceleration and maximal sprint capacity, while match external load data were subsequently collected during official matches of the 2024/2025 season (regular season and play-off phase). No experimental intervention, manipulation of training load, or modification of competitive conditions was implemented. All data were obtained as part of the regular monitoring processes conducted within the natural sporting environment. The study is reported following the STROBE guidelines for observational studies [30].

### 2.2. Participants

Data from male outfield football players with cerebral palsy or acquired brain injury holding a valid competitive licence from the Spanish Federation of Sports for Persons with Cerebral Palsy (FEDPC) and competing in the First Division of the Spanish National Football 7 League (seven teams) during the 2024/2025 season were analysed. Inclusion criteria were: an official sport class (FT1, FT2 or FT3) according to the classification system for CP football [7,19], completion of the pre-season sprint test, and at least one recorded official match. Goalkeepers were excluded. Each player retained the same sport class throughout the study period (no reclassification occurred). Sixty-three players were included in the analyses (age: 30.5 ± 8.8 years); playing position and impairment profile (spastic, ataxic or dyskinetic) were not systematically recorded in the dataset. The characteristics of the players included in the analyses are presented in Table 1.

A total of 285 player–match records were available. Observations were included when players accumulated at least 10 min of effective playing time (see Section 2.3); all records met this criterion. In addition, 12 observations with ACCDEC values above the upper Tukey fence (third quartile + 1.5 × interquartile range = 8.32 m·min^−1^; excluded values: 8.72–23.66 m·min^−1^) were considered implausible for this population, attributed to device recording errors, and excluded [31]; their influence was examined in a sensitivity analysis. The final dataset comprised 273 player–match observations from 63 players (FT1: 12 players and 47 observations; FT2: 38 and 167; FT3: 13 and 59) recorded in 28 official matches (200 regular-season and 73 play-off observations). The median number of observations per player was 5 (range 1–8) and was similar across classes (Table 1). All participants provided informed consent prior to participation, and the study was conducted in accordance with the principles of the Declaration of Helsinki. This study was conducted as part of a research project approved by the Ethics Committee of the Miguel Hernández University (approval number ADH.DES.IPG.JFM.24). The match data partially overlap with those used in previous studies by our group on match demands and team performance in the same league [6,22]; the present analysis addresses a different research question.

### 2.3. Procedures

Before the start of the competitive season (7–15 days before each team’s first official match), players completed an anthropometric assessment and a sprint test. Body mass and height were measured using a digital scale (Tanita BC-601, Tanita Corp., Tokyo, Japan; precision 0.1 kg) and a fixed stadiometer (SECA, Hamburg, Germany; precision 0.1 cm), respectively. After a standardised warm-up (3 min of low-intensity running, 5 min of mobility and dynamic stretching, and 3 min of progressively more intense actions, including jumps, accelerations and changes in direction), players performed two maximal 30-m linear sprints on artificial turf wearing their usual football boots, starting from a standing position with the front foot 0.3 m behind the starting line, with 2 min of passive recovery between attempts [23]. Times were recorded with photoelectric cells (Witty System, Microgate, Bolzano, Italy) placed at 0, 5 and 30 m at approximately hip height (adjusted to each participant), and the best attempt was retained for analysis. Average sprint velocities over 5 m (V5) and 30 m (V30) were calculated by dividing the sprint distance (m) by the corresponding time (s) and expressed in m·s^−1^. All players included in the analyses completed the sprint test. Sprint capacity was assumed to remain stable throughout the season, except for one player who completed additional sprint tests at different points of the season, for whom the corresponding test values were used.

Match external load was recorded with a wearable device (OLI, OLIVER, Sport Data Innovation S.L., Barcelona, Spain) integrating a global navigation satellite system (GNSS) receiver sampling at 10 Hz and an inertial measurement unit (accelerometer and gyroscope) sampling at 50 Hz. Following the manufacturer’s recommendations, the device was placed in a calf sleeve on the posterior side of the dominant leg, at the level of the gastrocnemius muscle belly; devices were fitted by the same researcher before each match and initialised in a static position [23,24]. This system has shown good agreement with other commercial tracking systems in futsal [32] and has discriminated match demands between competitive contexts and sport classes in CP football [6,22], although the validity of acceleration-based metrics obtained with a leg-worn device has not been specifically examined in players with asymmetric gait [33]. Data were processed with the manufacturer’s software using its default (proprietary) filtering and detection settings, including the minimum duration of accelerations and decelerations. The following variables were obtained: total distance covered (TD); high-intensity running distance (HI_running), defined as the distance covered above 18 km·h^−1^; and the distance covered during high-intensity acceleration and deceleration (ACCDEC), defined as the distance covered while accelerating above 3 m·s^−2^ or decelerating below −3 m·s^−2^ [22,23]. Because the device recorded continuously while worn, the recorded time included the half-time interval and, in play-off matches that went to extra time, the additional time associated with extra time. Effective playing time was therefore estimated by subtracting 10 min from recorded times longer than 40 min (half-time interval) and a further 10 min from play-off recorded times longer than 80 min (extra time); regular-season matches have no extra time and were not adjusted for it. All per-minute variables were calculated from the total distances divided by effective playing time.

Two distinct normalisation steps were applied. First, to account for differences in match exposure, TD, HI_running and ACCDEC were divided by each player’s effective playing time in that match, yielding per-minute values (m·min^−1^). Second, to express match demands relative to individual capacity, each per-minute variable was divided by V5 or V30, yielding six demand–capacity indices: TD/V5, TD/V30, HI/V5, HI/V30, ACCDEC/V5 and ACCDEC/V30. Because the numerator is expressed in m·min^−1^ and the denominator in m·s^−1^, the indices are expressed in s·min^−1^; for example, ACCDEC/V5 corresponds to the time (s) per minute of play that the player would need, at his average 5-m sprint velocity, to cover the distance he covered while accelerating or decelerating at high intensity. For the same ACCDEC, a higher ACCDEC/V5 value reflects lower acceleration capacity, whereas for the same V5 it reflects a greater acceleration–deceleration demand. The indices are therefore comparative descriptors of external demand relative to measured sprint capacity and do not directly quantify physiological effort, fatigue or internal load. A ratio was used, rather than individualised thresholds (e.g., accelerations expressed relative to each player’s maximal acceleration [16]), because it can be derived from routinely collected sprint tests and match reports; its validity, however, depends on the proportionality between numerator and denominator, which was tested explicitly (Section 2.4). ACCDEC/V5 was defined a priori as the index of primary interest, on the conceptual grounds described in the Introduction (the correspondence between short, high-intensity speed changes and short-distance acceleration capacity); the remaining indices are reported descriptively.

### 2.4. Statistical Analysis

Statistical analyses were performed in R (version 4.3.3; R Foundation for Statistical Computing, Vienna, Austria) [34] using the lme4, lmerTest and emmeans packages [35,36,37]. Descriptive statistics are reported as mean ± standard deviation, at player level for anthropometric and sprint variables (one value per player) and at observation level for match variables. Because players contributed a variable number of observations (Table 1), all inferential analyses accounted for the repeated-measures structure of the data [38,39]. Statistical significance was set at *p* < 0.05.

Associations among match variables, sprint variables and indices were examined in two ways. Between-player associations were quantified with Pearson’s correlation coefficients calculated on player means (n = 63). Within-player associations among the raw match variables were quantified with repeated-measures correlations (rrm) [40]; within-player correlations between an index and its numerator were not computed because V5 and V30 were constant within each player (except for the player with several sprint tests), so that each index is a fixed rescaling of its numerator within players (rrm = 1.00 by construction). Correlations were interpreted as trivial (<0.10), small (0.10–0.29), moderate (0.30–0.49), large (0.50–0.69), very large (0.70–0.89), and nearly perfect (≥0.90) [41]. The appropriateness of ratio scaling was assessed following Allison et al. [28] and Curran-Everett [29]: player mean ACCDEC was regressed on V5, and the ratio was considered appropriate if the intercept did not differ from zero (i.e., the relationship passed through the origin) and the resulting index was uncorrelated with V5.

Linear mixed-effects models were fitted by restricted maximum likelihood with random intercepts for player and team (players nested within teams) and competition phase (regular season vs. play-off), the only match-context variable available for every observation, as a fixed covariate. Four models were fitted: M1, ACCDEC with sport class as fixed effect; M2, ACCDEC/V5 with sport class; M3, ACCDEC with V5 (player-level, grand-mean centred) and sport class; and M4, ACCDEC with V5 only. Neither ACCDEC nor V5 was entered as a predictor of the ratio outcome, to avoid the mathematical coupling between outcome and covariate that arises when a ratio is adjusted for its own numerator [27,29]. Models M3 and M4 tested, on the raw variables, whether V5 was associated with ACCDEC and whether sport class explained ACCDEC beyond V5. Fixed effects were tested with F-tests using Satterthwaite’s approximation for the degrees of freedom; the contributions of V5 and sport class were additionally examined with likelihood-ratio tests on maximum-likelihood fits. Estimated marginal means (EMMs) with 95% confidence intervals (CIs) were obtained for each class, and pairwise comparisons were Holm-adjusted [42]. Marginal (R^2^m, fixed effects) and conditional (R^2^c, fixed and random effects) coefficients of determination were calculated [43]. Residuals were inspected with quantile–quantile plots; because they were positively skewed, models were refitted with log-transformed ACCDEC as a sensitivity check. The smallest worthwhile change (SWC) was estimated as 0.2 × the between-player standard deviation [41].

Inter-individual variability within each class was described with the coefficient of variation (CV) of ACCDEC and ACCDEC/V5 across observations, with 95% CIs obtained by player-level cluster bootstrap (2000 resamples, percentile method). Three sensitivity analyses repeated models M1–M4 (i) including only observations with ≥30 min of effective playing time, to reduce the noise of per-minute values derived from short appearances, (ii) retaining the 12 extreme ACCDEC values, and (iii) applying the extra-time correction also to regular-season observations with recorded times longer than 80 min. Figure 1 and Figure 2 are descriptive: in Figure 1, players were grouped into tertiles of V5 (cut-offs: 3.85 and 4.17 m·s^−1^) for display only, and in Figure 2 the median ACCDEC (observation level) and the median V5 (player level) were used as reference lines; these categories were not used in any inferential analysis. No a priori sample size calculation was performed because all available league data were analysed; with 63 players, between-player correlations of |r| ≥ 0.35 could be detected with 80% power (α = 0.05).

## 3. Results

### 3.1. Relationships Among Match External Load Variables, Sprint Capacity and Demand–Capacity Indices

Between-player correlations are presented in Table 2. TD showed a very large correlation with HI_running (r = 0.81) and a small, non-significant correlation with ACCDEC (r = 0.13, 95% CI −0.13 to 0.36), whereas HI_running and ACCDEC were moderately correlated (r = 0.35). Within players, repeated-measures correlations were large between TD and HI_running (rrm = 0.57, 95% CI 0.47 to 0.66) and moderate between TD and ACCDEC (rrm = 0.36, 0.23 to 0.47) and between HI_running and ACCDEC (rrm = 0.41, 0.29 to 0.52). V5 and V30 were nearly perfectly correlated (r = 0.91), and both were moderately correlated with TD, HI_running and ACCDEC (r = 0.30 to 0.44).

Each index was correlated with its numerator: very largely for the TD-based indices (r = 0.72 to 0.75) and nearly perfectly for the HI_running- and ACCDEC-based indices (r = 0.96 to 0.97). TD-based indices were negatively correlated with V5 and V30 (r = −0.26 to −0.36), whereas HI_running- and ACCDEC-based indices were not significantly correlated with either sprint variable (r = 0.08 to 0.23). ACCDEC/V5 showed trivial correlations with TD and the TD-based indices (r = −0.06 to 0.04) and small correlations with HI_running and the HI_running-based indices (r = 0.24 to 0.26). Within players, ACCDEC/V5 is a constant rescaling of ACCDEC (rrm = 1.00).

### 3.2. Association Between ACCDEC and Acceleration Capacity

At player level, mean ACCDEC increased with V5 (slope = 1.11 m·min^−1^ per m·s^−1^, 95% CI 0.22 to 1.99; *p* = 0.015), and the intercept did not differ from zero (−0.52 m·min^−1^, 95% CI −4.06 to 3.01; *p* = 0.769), indicating that the relationship was compatible with proportionality. Consistently, ACCDEC/V5 was not correlated with V5 (r = 0.08, 95% CI −0.17 to 0.32; *p* = 0.54). In the mixed model including all observations (M4), higher V5 was associated with greater ACCDEC (B = 0.91 m·min^−1^ per m·s^−1^, 95% CI 0.11 to 1.71; F(1, 61.3) = 4.98; *p* = 0.029; R^2^m = 0.04, R^2^c = 0.51). Between-player differences accounted for 49% of the variance in ACCDEC, team for less than 1%, and within-player (match-to-match) variation for 51%. Figure 1 shows the relationship between ACCDEC and ACCDEC/V5. The index increased linearly with ACCDEC, and for a given ACCDEC, observations from players with lower V5 showed higher index values, as follows from the construction of the ratio.

### 3.3. Demand–Capacity Profiles

Figure 2 plots V5 against ACCDEC for each observation, with the median ACCDEC (3.67 m·min^−1^) and the median V5 (4.07 m·s^−1^) as reference lines. The upper-right quadrant contains observations with high demand and high acceleration capacity, the upper-left quadrant low demand and high capacity, the lower-left quadrant low demand and low capacity, and the lower-right quadrant high demand relative to low capacity. Observations from all sport classes were found in all four quadrants. Most FT1 observations (79%) were located at or below the median V5, and 23% were in the high-demand/low-capacity quadrant, whereas 41% of FT3 observations were in the high-demand/high-capacity quadrant. These quadrants are descriptive, depend on the sample medians, and were not tested statistically.

### 3.4. Differences Between Sport Classes

Estimated marginal means are presented in Table 3 and Figure 3. The effect of sport class on ACCDEC was borderline (M1: F(2, 48.6) = 3.06; *p* = 0.056; R^2^m = 0.06, R^2^c = 0.52), with FT1 players tending to accumulate less ACCDEC than FT3 players (difference −1.25 m·min^−1^, Holm-adjusted 95% CI −2.50 to 0.00; *p* = 0.050); the FT1–FT2 (−0.66 m·min^−1^; *p* = 0.247) and FT2–FT3 (−0.59 m·min^−1^; *p* = 0.247) differences were not significant. In contrast, sport class had no significant effect on ACCDEC/V5 (M2: F(2, 50.6) = 1.22; *p* = 0.304; R^2^m = 0.03, R^2^c = 0.54), with between-class differences of 0.09 to 0.21 s·min^−1^ and confidence intervals including zero (Table 3). Competition phase was not associated with either outcome (*p* ≥ 0.48).

When V5 and sport class were entered together (M3), neither V5 (B = 0.64 m·min^−1^ per m·s^−1^, 95% CI −0.20 to 1.49; F(1, 58.5) = 2.23; *p* = 0.140) nor sport class (F(2, 49.2) = 1.61; *p* = 0.211) was significant, reflecting their shared variance (V5 differed between classes; Table 1). Adding sport class to a model containing V5 did not improve fit (χ^2^(2) = 3.00; *p* = 0.223), nor did adding V5 to a model containing sport class (χ^2^(1) = 2.34; *p* = 0.126). After adjustment for V5, the FT1–FT3 difference in ACCDEC decreased from −1.25 to −0.96 m·min^−1^ (*p* = 0.241).

Within-class variability of ACCDEC/V5 decreased from FT1 (CV = 52.7%, 95% CI 35.9 to 60.6%) to FT2 (39.5%, 34.3 to 44.5%) and FT3 (33.5%, 25.6 to 41.6%). A similar pattern was observed for ACCDEC itself (FT1: 48.9%, 35.6 to 56.9%; FT2: 38.8%, 33.6 to 43.6%; FT3: 32.3%, 24.0 to 39.7%), and the confidence intervals overlapped between classes.

Residuals of all models were positively skewed (skewness ≈ 0.8). With log-transformed ACCDEC, V5 was significantly associated with ACCDEC in M3 (*p* = 0.010), whereas sport class was not (*p* = 0.273). When the analyses were restricted to observations with ≥30 min of effective playing time (256 observations, 57 players), the effect of sport class was not significant for ACCDEC (F(2, 49.0) = 1.93; *p* = 0.156) or ACCDEC/V5 (F(2, 51.0) = 0.40; *p* = 0.673), and V5 was not associated with ACCDEC (M4: *p* = 0.446). Retaining the 12 extreme values (285 observations), neither sport class (ACCDEC: F(2, 61.1) = 2.06, *p* = 0.136; ACCDEC/V5: F(2, 60.0) = 0.96, *p* = 0.388) nor V5 (M4: *p* = 0.277) was significant. Applying the extra-time correction also to regular-season observations yielded virtually identical results to the main analysis (sport class on ACCDEC: *p* = 0.059; ACCDEC/V5: *p* = 0.305; V5 in M4: *p* = 0.027). The SWC was 0.30 m·min^−1^ for ACCDEC and 0.076 s·min^−1^ for ACCDEC/V5.

## 4. Discussion

The aims of this exploratory study were to examine how ACCDEC relates to acceleration capacity in CP football, whether a demand–capacity ratio (ACCDEC/V5) is statistically appropriate, and how both variables differ between sport classes. Three main findings emerged. First, ACCDEC/V5 was nearly identical to ACCDEC between players and, by construction, identical within players, so the only additional information it carries is V5. Second, ACCDEC increased with V5 and the relationship was compatible with proportionality, so the ratio was uncorrelated with V5, which is the condition under which ratio scaling removes the influence of the denominator [28,29]. Third, FT1 players tended to accumulate less ACCDEC than FT3 players, whereas no class differences were observed for ACCDEC/V5 and the class effect on ACCDEC was further attenuated when V5 was taken into account; however, these class effects were small, borderline, and not robust in sensitivity analyses. Taken together, the findings support ACCDEC/V5 as a descriptive, capacity-scaled expression of acceleration–deceleration demands, but not as a measure of functional or physiological cost.

Among the different indices, ACCDEC/V5 was the index of primary interest because accelerations and decelerations involve rapid force production, braking capacity, and postural control [44,45,46], functions that are frequently affected in players with CP [21,47,48], and because the reduced pitch dimensions of CP football limit sustained high-speed running and place greater emphasis on short, high-intensity actions. Short-distance acceleration performance has been linked to the neuromuscular factors that underpin explosive actions in intermittent sports [49,50], so V5 is the capacity measure that most closely matches this type of demand. Conversely, indices based on total distance or high-intensity running primarily reflect overall locomotor volume and are strongly influenced by contextual and tactical factors [13,51,52]; in addition, TD-based indices were negatively correlated with sprint capacity (Table 2), indicating that dividing TD by V5 or V30 over-corrects for capacity.

The nearly perfect correlation between ACCDEC and ACCDEC/V5 must be interpreted in light of the mathematical coupling between a ratio and its numerator [27,28,29]. Because V5 was measured once for all but one player, ACCDEC/V5 is a fixed rescaling of ACCDEC within each player, and, between players, differences in the index at comparable ACCDEC values (Figure 1) necessarily reflect differences in V5. Thus, the vertical dispersion observed in Figure 1 should not be interpreted as independent evidence that similar demands impose different costs, but as a representation of the combination of two measured variables. Whether expressing them as a single ratio adds practical value beyond reporting ACCDEC and V5 together cannot be established with the present data, as no external criterion, such as internal load, neuromuscular fatigue or performance, was available [14,25].

Players with higher V5 accumulated more ACCDEC per minute, although the association was moderate between players (r = 0.30), explained a small proportion of the observation-level variance (R^2^m = 0.04), and was not observed when the analysis was restricted to observations with ≥30 min of effective playing time. This is consistent with Maggiolo et al. [24], who reported limited associations between sprint tests and match variables in international CP footballers, and indicates that most of the variability in ACCDEC was attributable to other between-player factors and to match-to-match fluctuations (51% of the variance). Tactical role, playing position, opponent quality, and match status are known to influence external load [10,12,13,51,53], and playing position has been shown to affect match demands and perceived exertion in CP football [25]; these factors could not be assessed at observation level in the present dataset and probably account for part of the unexplained variance. The proportionality finding has a practical implication: because the intercept of the ACCDEC–V5 relationship did not differ from zero and the index was unrelated to V5, ACCDEC/V5 can be used to compare players with different acceleration capacities without the scaling bias that affects ratios when this condition is violated [28,29]. However, the confidence interval of the intercept was wide, so this conclusion should be confirmed in larger samples.

The quadrant representation in Figure 2 offers a simple way to visualise combinations of match demand and acceleration capacity. Because the cut-points are sample medians rather than externally validated thresholds, they would shift in another cohort; the qualitative pattern, namely that observations with high ACCDEC in players with low V5 represent high demands relative to capacity, is therefore more robust than the specific boundaries. Whether this profile is associated with greater neuromuscular strain, fatigue or injury risk, as suggested for repeated high-intensity decelerations in other team sports [46,54], is a hypothesis that requires internal-load or neuromuscular measures, which are feasible in CP football [25].

FT1 players tended to accumulate approximately 1.2 m·min^−1^ less ACCDEC than FT3 players (*p* = 0.056 for the overall class effect), in line with previous reports of lower high-intensity output in more impaired classes [8,9,21]. When ACCDEC was expressed relative to, or adjusted for, V5, class differences were further attenuated. A plausible interpretation is that part of the between-class difference in acceleration–deceleration output reflects differences in acceleration capacity, which is itself an activity-limitation outcome that differs between classes [20,26]. Impairment-related constraints in selective motor control, inter-limb coordination, balance, and braking capacity may contribute to these differences [45,47,48], but these mechanisms were not measured and remain hypotheses. The absence of a significant class effect on ACCDEC/V5 should not be interpreted as equivalence between classes, given the wide confidence intervals. Moreover, the class effect on ACCDEC itself was borderline and weaker when short appearances or extreme values were handled differently, probably because of the small number of FT1 and FT3 players (12 and 13, respectively).

Similarly, the progressively lower coefficients of variation from FT1 to FT3 were almost identical for ACCDEC and ACCDEC/V5, and their bootstrap confidence intervals overlapped. This indicates that they mainly reflect the within-class variability of ACCDEC, together with the imprecision associated with the small number of FT1 players, rather than a property of the index. The greater heterogeneity of impairment presentations and compensatory movement strategies in players with more severe impairment [55] might contribute to this pattern, but this cannot be confirmed with the present data.

### Strengths and Limitations

The main strengths of this study are the use of official match data from an entire national league season, the use of mixed-effects models that account for repeated observations of the same players and their clustering within teams, the explicit testing of the assumptions underlying ratio scaling, and the inclusion of sensitivity analyses. Several limitations should, however, be acknowledged. First, as an observational study, contextual factors such as playing position, tactical role, opponent quality, and match status were not recorded at observation level, and competition phase was the only match-context variable available. Second, no internal-load, physiological, or neuromuscular measures were collected, so the index could not be validated as a measure of effort or cost. Third, because of the mathematical coupling between the index and its numerator, ACCDEC/V5 adds no information beyond ACCDEC and V5 considered jointly. Fourth, sprint capacity was measured before the season and applied to all subsequent matches (except for one player retested during the season), so within-season changes due to training adaptation, fatigue, or fluctuations related to the players’ condition were not captured; regular (e.g., weekly) reassessment of V5 would address this limitation (Section 5). Fifth, because the device recorded continuously, effective playing time had to be estimated by subtracting fixed durations for half-time and extra time, which may have introduced some error in per-minute values. Sixth, the device was worn on the leg, and the validity of acceleration-based metrics obtained in this position has not been established in players with asymmetric or ataxic gait; measurement error could therefore differ between classes. Seventh, the sample was a convenience sample with few FT1 and FT3 players, which limited the precision of between-class comparisons; additionally, 12 extreme observations were excluded, although a sensitivity analysis retaining them led to similar conclusions. Eighth, players were grouped by sport class, but within-class differences in impairment type and neuromotor mechanisms (e.g., spasticity pattern, coordination, or selective motor control), which may influence acceleration–deceleration demands, could not be distinguished. Finally, acceleration capacity was assessed with a linear sprint test using single-beam photocells whose test–retest reliability could not be determined in this sample (only the best attempt was retained), so 5-m times may include some measurement error; in addition, a linear test may not capture the multidirectional and reactive nature of match play, and the findings, derived from male national-level players, may not generalise to other competitive levels or to female CP football.

## 5. Practical Applications

For practitioners, the present results suggest reporting ACCDEC together with each player’s V5, or as the ACCDEC/V5 ratio, when comparing players with different acceleration capacities or sport classes. Expressed in this way, a given ACCDEC value can be read as high or low relative to what the player is able to produce in a short sprint, which may help to contextualise individual match reports. Because the index is a constant rescaling of ACCDEC within each player, it adds no information when monitoring the same player across matches unless V5 is reassessed; its potential use lies in comparisons between players. Differences smaller than the SWC (approximately 0.3 m·min^−1^ for ACCDEC and 0.08 s·min^−1^ for ACCDEC/V5) should not be interpreted as meaningful.

In addition, the index could be expressed relative to each player’s current capacity by assessing 5-m sprint performance regularly (e.g., weekly, in a training session close to competition) rather than only before the season. Because the 5-m sprint is brief, requires minimal equipment and induces little fatigue, it could be integrated into routine training, so that each match observation is expressed relative to an up-to-date measure of acceleration capacity. This would remove the assumption of stable capacity throughout the season and would allow the index to be used for monitoring the same player across matches, as changes in V5 would then be reflected in the index. Before this approach is adopted, however, the reliability and sensitivity of repeated V5 assessments in CP football players should be established.

Training programmes aimed at improving short-distance acceleration and braking capacity are a reasonable target in CP football, given the importance of short, high-intensity actions on a reduced pitch. However, whether improving V5 changes match ACCDEC, or its physiological consequences, was not tested in the present study and should be examined in longitudinal or intervention studies.

Regarding classification, evidence-based classification in Para sport requires measures of activity limitation that are valid, reliable, and resistant to training and intentional misrepresentation [56]. The present data do not support the use of ACCDEC/V5 in classification: the index is largely a function of V5, itself a test outcome that differs between classes, so using it to allocate players to classes would be partly circular, and it has not been validated against classification outcomes or panel decisions.

Nevertheless, the findings raise questions that future research could address to inform the refinement of the classification system. For example, studies could examine whether demand–capacity profiles collected during the observation-in-competition phase provide information beyond the activity-limitation tests that currently best predict sport class, such as dribbling speed, inter-limb coordination, and static balance [26]; whether players whose match profiles differ markedly from those expected for their class could be identified for review; and whether such measures could help to evaluate the extent to which the current three-class system groups players with similar match-related activity limitation. Answering these questions would require larger, multinational samples, validation against classification panel decisions, and evidence of reliability before any practical adoption in classification settings.

## 6. Conclusions

In CP football players from the Spanish National League, (i) ACCDEC was positively but moderately associated with acceleration capacity (V5); (ii) the ACCDEC–V5 relationship was compatible with proportionality, so ACCDEC/V5 provides a capacity-scaled expression of acceleration–deceleration demands that is not biased by V5, but it adds no information beyond ACCDEC and V5 considered jointly and should not be interpreted as a measure of functional or physiological cost; and (iii) FT1 players tended to accumulate less ACCDEC than FT3 players, but this difference was borderline, was not observed for ACCDEC/V5, and was not robust in sensitivity analyses. Future studies should validate demand–capacity indices against internal-load, neuromuscular, or performance criteria before recommending their use for load monitoring or classification.

## Figures and Tables

**Figure 1:**
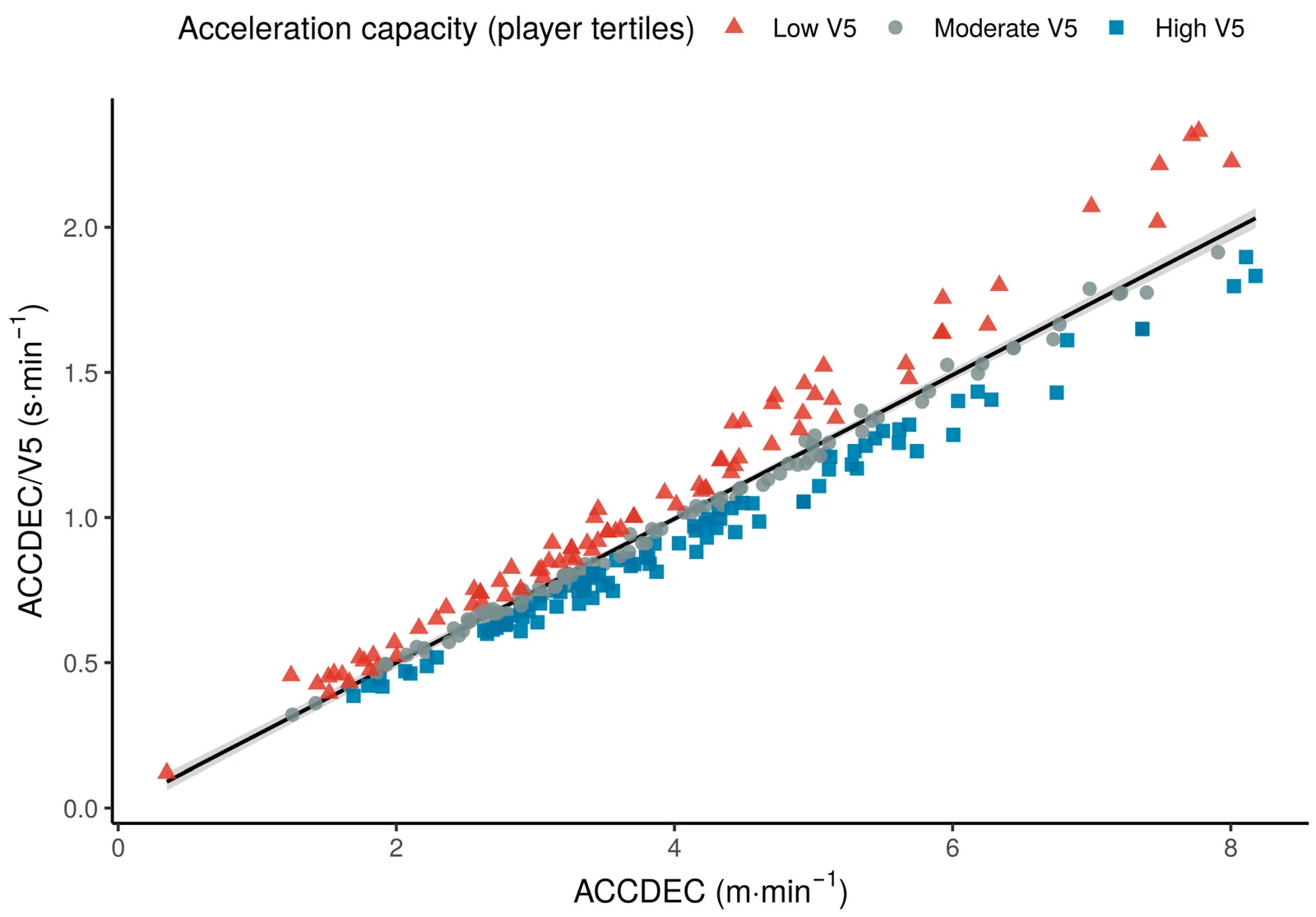
Relationship between the distance covered during high-intensity accelerations and decelerations per minute (ACCDEC) and the ACCDEC/V5 demand–capacity index (273 observations from 63 players). Each point represents a player–match observation. Colours and marker shapes indicate tertiles of acceleration capacity (V5) computed at player level (Low: ≤3.85 m·s^−1^; Moderate: >3.85 to 4.17 m·s^−1^; High: >4.17 m·s^−1^), used for display purposes only. The black line and shaded band show the linear trend and its 95% confidence interval. Because V5 is constant within each player (except one), the vertical separation of the tertiles at a given ACCDEC follows arithmetically from the construction of the index.

**Figure 2:**
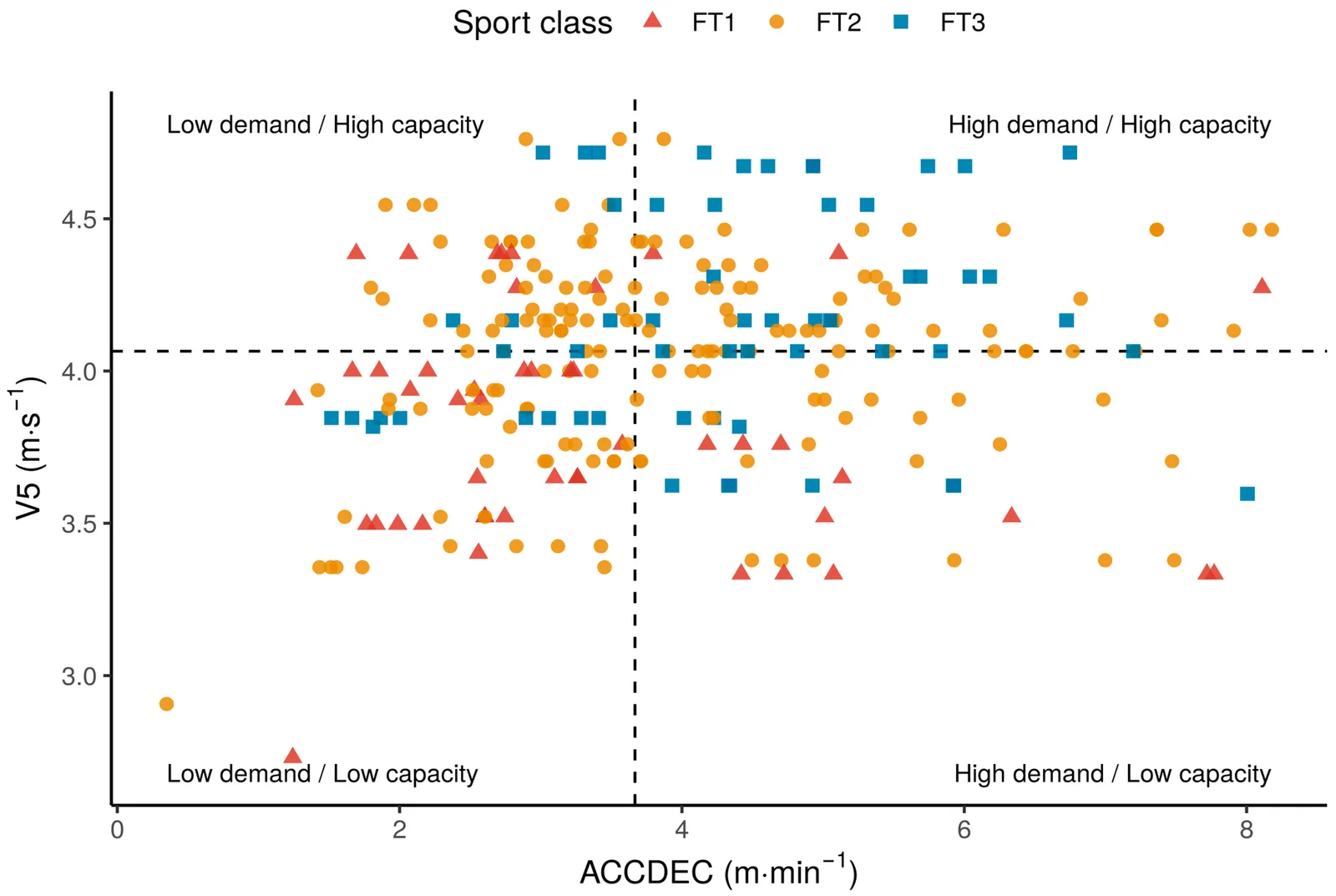
Relationship between the distance covered during high-intensity accelerations and decelerations per minute (ACCDEC) and acceleration capacity (average 5-m sprint velocity, V5). Each point represents a player–match observation; colours and marker shapes indicate sport class. Dashed lines show the median ACCDEC (observation level) and the median V5 (player level), dividing the plot into four descriptive quadrants: high demand/high capacity (upper right), low demand/high capacity (upper left), low demand/low capacity (lower left), and high demand/low capacity (lower right). Cut-points are sample medians and would differ in other cohorts.

**Figure 3:**
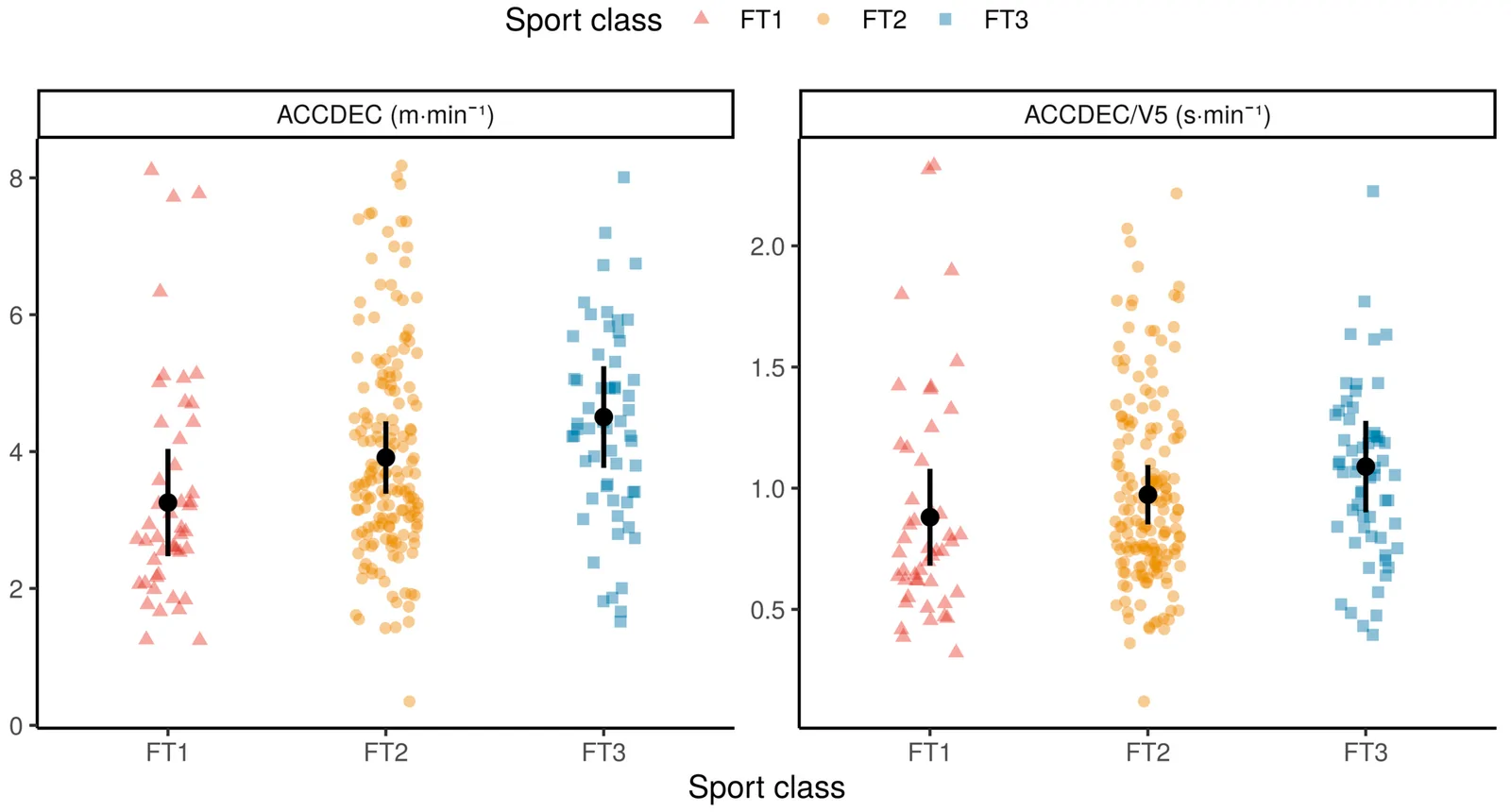
Distance covered during high-intensity accelerations and decelerations per minute (ACCDEC; (**left panel**)) and ACCDEC/V5 demand–capacity index (**right panel**) by sport class. Small symbols represent individual player–match observations (horizontally jittered); black circles and bars represent estimated marginal means and 95% confidence intervals from linear mixed-effects models with random intercepts for player and team, adjusted for competition phase (models M1 and M2).

**Table 1:** Characteristics of the players (player level) and match external load variables (observation level) by sport class.

Variable	FT1	FT2	FT3	Overall
Players (n)	12	38	13	63
Observations (n)	47	167	59	273
Observations per player, median (range)	4 (1–7)	5 (1–8)	5 (1–7)	5 (1–8)
Body mass (kg)	66.24 ± 7.08	69.83 ± 8.04	61.92 ± 6.94	67.47 ± 8.16
Height (m)	1.77 ± 0.05	1.76 ± 0.06	1.72 ± 0.09	1.75 ± 0.07
V5 (m·s^−1^)	3.70 ± 0.45	4.00 ± 0.38	4.11 ± 0.37	3.97 ± 0.41
V30 (m·s^−1^)	5.43 ± 0.67	6.04 ± 0.50	6.18 ± 0.37	5.95 ± 0.57
*Observation level*				
Effective playing time (min)	51.36 ± 19.03	57.84 ± 16.06	58.62 ± 14.51	56.89 ± 16.43
TD (m·min^−1^)	83.61 ± 17.40	90.45 ± 13.03	95.78 ± 11.71	90.42 ± 14.08
HI_running (m·min^−1^)	8.26 ± 3.70	10.37 ± 4.47	13.72 ± 4.97	10.73 ± 4.78
ACCDEC (m·min^−1^)	3.38 ± 1.65	3.96 ± 1.54	4.37 ± 1.41	3.95 ± 1.55
ACCDEC/V5 (s·min^−1^)	0.90 ± 0.48	0.98 ± 0.39	1.05 ± 0.35	0.98 ± 0.40
ACCDEC/V30 (s·min^−1^)	0.62 ± 0.33	0.65 ± 0.26	0.70 ± 0.22	0.65 ± 0.26

Values are mean ± standard deviation unless otherwise indicated. Player-level variables (body mass, height, V5, V30) were computed once per player; match variables were computed across player–match observations. Body mass and height were available for 52 players (FT1: 10; FT2: 31; FT3: 11). Match variables are expressed per minute of effective playing time. TD = total distance covered per minute; HI_running = high-intensity running distance (>18 km·h^−1^) per minute; ACCDEC = distance covered during high-intensity accelerations (>3 m·s^−2^) and decelerations (<−3 m·s^−2^) per minute; V5 = average sprint velocity over 5 m; V30 = average sprint velocity over 30 m. FT1–FT3 = official sport classes in cerebral palsy football.

**Table 2:** Between-player Pearson’s correlation coefficients (r) among match external load variables, sprint capacity, and demand–capacity indices (player means; n = 63 players).

Variable	1	2	3	4	5	6	7	8	9	10
1. TD (m·min^−1^)	—									
2. HI_running (m·min^−1^)	0.81 *	—								
3. ACCDEC (m·min^−1^)	0.13	0.35 *	—							
4. V5 (m·s^−1^)	0.37 *	0.43 *	0.30 *	—						
5. V30 (m·s^−1^)	0.38 *	0.44 *	0.32 *	0.91 *	—					
6. TD/V5 (s·min^−1^)	0.72 *	0.49 *	−0.11	−0.36 *	−0.29 *	—				
7. TD/V30 (s·min^−1^)	0.75 *	0.50 *	−0.12	−0.26 *	−0.32 *	0.95 *	—			
8. HI/V5 (s·min^−1^)	0.80 *	0.96 *	0.30 *	0.19	0.23	0.66 *	0.64 *	—		
9. HI/V30 (s·min^−1^)	0.80 *	0.97 *	0.29 *	0.23	0.23	0.63 *	0.65 *	0.99 *	—	
10. ACCDEC/V5 (s·min^−1^)	0.04	0.25	0.97 *	0.08	0.12	−0.03	−0.06	0.26 *	0.24	—
11. ACCDEC/V30 (s·min^−1^)	0.03	0.24	0.97 *	0.12	0.10	−0.07	−0.07	0.24	0.23	0.99 *

* *p* < 0.05. Within-player repeated-measures correlations: TD–HI_running, rrm = 0.57; TD–ACCDEC, rrm = 0.36; HI_running–ACCDEC, rrm = 0.41 (all *p* < 0.001; 209 degrees of freedom). Within players, each index is a constant rescaling of its numerator (rrm = 1.00). Abbreviations as in Table 1.

**Table 3:** Estimated marginal means and pairwise differences between sport classes (95% confidence intervals) from linear mixed-effects models.

	ACCDEC (m·min^−1^), M1	ACCDEC/V5 (s·min^−1^), M2	ACCDEC Adjusted for V5 (m·min^−1^), M3
FT1	3.26 (2.47, 4.04)	0.88 (0.68, 1.08)	3.46 (2.65, 4.26)
FT2	3.91 (3.38, 4.44)	0.97 (0.85, 1.10)	3.93 (3.44, 4.42)
FT3	4.51 (3.76, 5.25)	1.09 (0.90, 1.28)	4.41 (3.69, 5.14)
FT1 − FT2	−0.66 (−1.70, 0.38); *p* = 0.247	−0.09 (−0.37, 0.19); *p* = 0.570	−0.47 (−1.56, 0.62); *p* = 0.476
FT1 − FT3	−1.25 (−2.50, 0.00); *p* = 0.050	−0.21 (−0.54, 0.13); *p* = 0.383	−0.96 (−2.28, 0.37); *p* = 0.241
FT2 − FT3	−0.59 (−1.58, 0.40); *p* = 0.247	−0.12 (−0.38, 0.15); *p* = 0.570	−0.48 (−1.49, 0.52); *p* = 0.476
Class effect	F(2, 48.6) = 3.06; *p* = 0.056	F(2, 50.6) = 1.22; *p* = 0.304	F(2, 49.2) = 1.61; *p* = 0.211

All models included random intercepts for player and team and competition phase as a fixed covariate (marginal means averaged over phases); M3 additionally included V5 (grand-mean centred). Degrees of freedom by Satterthwaite’s approximation; pairwise comparisons Holm-adjusted (confidence intervals adjusted for three comparisons); 273 observations from 63 players. Abbreviations as in Table 1.

## Data Availability

The data supporting the findings of this study are available from the corresponding author upon reasonable request. The data are not publicly available due to privacy and ethical considerations related to the protection of participants’ personal and performance data.
